# Impact of Parenteral Ceftiofur on Developmental Dynamics of Early Life Fecal Microbiota and Antibiotic Resistome in Neonatal Lambs

**DOI:** 10.3390/antibiotics14050434

**Published:** 2025-04-25

**Authors:** Mohamed Donia, Nasr-Eldin Aref, Mohamed Zeineldin, Ameer Megahed, Benjamin Blair, James Lowe, Brian Aldridge

**Affiliations:** 1 Department of Pathobiology, College of Veterinary Medicine, University of Illinois at Urbana-Champaign, Urbana-Champaign, IL 61802, USA; mdonia2@illinois.edu; 2 Department of Animal Medicine (Internal Medicine), Faculty of Veterinary Medicine, Kafrelsheikh University, Kafrelsheikh 33516, Egypt; 3 Department of Animal Medicine (Internal Medicine), Faculty of Veterinary Medicine, Assiut University, Assiut 71526, Egypt; nasreldeen.aref@vet.au.edu.eg; 4 Department of Animal Medicine (Internal Medicine), Faculty of Veterinary Medicine, Benha University, Moshtohor-Toukh 13736, Egypt; mohamed.zynaldeen@fvtm.bu.edu.eg (M.Z.); ameermegahed@ufl.edu (A.M.); 5 Department of Large Animal Clinical Sciences, College of Veterinary Medicine, University of Florida, Gainesville, FL 32610, USA; 6 Integrated Food Animal Management Systems, Department of Veterinary Clinical Medicine, College of Veterinary Medicine, University of Illinois at Urbana-Champaign, Urbana-Champaign, IL 61802, USA; bblair2@illinois.edu (B.B.); jlowe@illinois.edu (J.L.); 7 Department of Veterinary Medicine and Sciences, College of Veterinary Medicine, Clemson University, Clemson, SC 29634, USA

**Keywords:** microbiome, lambs, ceftiofur, antibiotic resistance

## Abstract

**Background:** Early gut microbiome development is critical for neonatal health, and its dysbiosis may impact long-term animal productivity. This study examined the effects of parenteral Ceftiofur Crystalline Free Acid (CCFA) on the composition and diversity of the neonatal lamb fecal microbiome. The emergence of antimicrobial resistance genes associated with CCFA exposure was also investigated. **Results:** There were distinct microbial populations in the CCFA-treated lambs compared to the control group at each time point, with a highly significant decrease in alpha and beta diversity. The CCFA treatment showed a reduction in several key microbial taxa during nursing, but these differences were diminished by day 56. Unlike the control group, CCFA-treated lambs had core microbes potentially carrying multiple antibiotic resistance genes, including those for beta-lactam, fosfomycin, methicillin, and multidrug resistance. **Methods:** Twenty-four healthy neonatal lambs were randomly assigned to CCFA-treated (n = 12) and control (n = 12) groups. Fecal samples were collected on days 0, 7, 14, 28, and 56. Genomic DNA was extracted and sequenced using the Illumina MiSeq platform. Microbial composition was analyzed using the MG-RAST pipeline with the RefSeq database. **Conclusions:** Despite temporary reductions in critical bacterial populations during nursing, the early sheep fecal microbiome demonstrated resilience by repopulating after CCFA antibiotic disruption. While this highlights microbiota stability after short-course antibiotic exposure, the transient disturbance underscores potential risks to early gut health. Importantly, persistent CCFA resistance poses environmental dissemination risks, emphasizing the need for cautious antibiotic use in livestock to mitigate ecological impacts.

## 1. Introduction

The livestock gut microbiome is an ecosystem with diverse microbial communities living in symbiosis with their host [1]. Homeostasis of this ecosystem is crucial in maintaining the host’s immune health and productivity [2]. Gut microbiota represents an innate barrier that protects against pathogen invasion and supplies the host with different metabolic products with nutritional, immune-modulatory, and antimicrobial properties [3]. Several studies have demonstrated that altering this microbial community (dysbiosis) may lead to perturbations in metabolism, immune-competence, and chronic disease [4,5]. Therefore, understanding the gut microbiome composition and developmental dynamics is essential for maintaining animal health and production.

The early colonization of the neonatal gut microbiome is critical and influences the host phenotype [6]. However, its origin is a matter of debate. Regardless of the origin of the first-seeded microbes, their growth generates an anaerobic environment after utilizing the available oxygen. This promotes the development of anaerobic communities such as Bacteroides and Firmicutes [7,8]. This development progresses into niche-specific habitats across different GIT segments, each hosting distinct luminal and mucosal gut microbiota populations [9,10]. Maturation of the mucosal immune system co-occurs with microbiome development to ensure microbio-mucosal homeostasis [11]. After stabilization, studies suggest that resident microbial populations are stable and resilient to temporary perturbations if there are no dramatic changes in host health and diet. However, any hindrance to this classic development pattern during early life is thought to be associated with profound health consequences [12,13]. 

Beyond the influence of host genetics, microbial colonization in newborn lambs is shaped by maternal factors, early nutrition, and the surrounding environment [14]. At birth, lambs encounter microbes from maternal sources such as the vaginal canal, feces, and teat skin, each providing early exposure to beneficial bacteria, including *Lactobacillus* and *Bifidobacterium*. These microbes contribute to the early development of gut structure and immune competence [15]. Colostrum and milk also play a central role, delivering immunological components like antibodies and antimicrobial peptides and introducing microbial species that help establish the neonatal gut flora [16]. As lambs grow, environmental exposures to microbes from bedding, housing surfaces, and the air further increase the diversification of their microbiota [17]. Nutritional transitions, particularly the shift from milk to solid feed, promote the rise of anaerobic fermenters such as *Prevotella* and *Ruminococcus*, key contributors to short-chain fatty acid production and rumen maturation. However, these early colonization processes can be disrupted when nutrition is suboptimal, such as with low-quality milk replacers, or when hygiene is compromised [18,19]. This may result in microbial imbalances that favor pathogenic species, potentially hindering growth and health outcomes.

Additionally, chemotherapeutics such as antibiotics are essential factors that can disrupt microbial composition and promote the development of microbial strains carrying antibiotic resistance genes (ARG) [20]. Despite recent recommendations to optimize antibiotic use to mitigate the global threat of antimicrobial resistance, antibiotics continue to be widely utilized in livestock production systems for disease treatment and prophylaxis [21]. One of these heavily used antibiotics in the veterinary practice is ceftiofur, the only third-generation cephalosporin approved for use in livestock in the US. It is widely used to treat mammary gland, uterine, gastrointestinal, and respiratory infections [22,23]. Due to the widespread use of ceftiofur and its importance in human therapeutics, this study selected ceftiofur to examine its impact on neonatal lamb microbial disruption and potential resistance development. Multiple lines of evidence indicate that antibiotic administration can disrupt the structure and development of maternal and neonatal mucosal microbiota [24]. However, knowledge of the early-life impact of perinatal antibiotic administration on the prevalence of the antibiotic resistome and the establishment of early-life mucosal microbiota in sheep is lacking. Additionally, while the ruminant [4] and pig [25] fecal microbiota have been characterized in various conditions of health and disease, the early development of the gut microbiome in sheep has yet to be fully elucidated.

In this study, we hypothesized that early-life exposure to parenteral antibiotics disrupts the natural progression of gut microbiota development and promotes the emergence of antimicrobial resistance genes (ARGs) in neonatal lambs. Therefore, we investigated the impact of a single dose of Ceftiofur Crystalline-Free Acid (CCFA) on the fecal microbiome composition, diversity, and resistome during two critical developmental stages: the nursing period and the transition to grazing within the first two months of life. We utilized whole-genome shotgun metagenomic sequencing (WGSS) to characterize microbiota dynamics over time and assess microbial resilience following CCFA intervention.

## 2. Results

### 2.1. Impact of CCFA on the Lamb’s Body Weight

On Day 1, female lambs had an average body weight of 3.88 ± 0.19 kg in the control group and 4.09 ± 0.23 kg in the treated group. Male lambs averaged 3.74 ± 0.28 kg in the control group and 4.12 ± 0.30 kg in the treated group. By Day 56, average weights for female lambs reached 26.60 ± 0.97 kg (control) and 27.10 ± 1.21 kg (treated), while male lambs reached 25.30 ± 1.30 kg (control) and 27.60 ± 1.36 kg (treated). Although males tended to be slightly heavier than females by Day 56, no statistically significant differences in body weight were detected between the sexes or treatment groups during the experiment.

### 2.2. Whole Genome Shotgun Sequencing Summary

Whole-genome shotgun metagenomic sequencing generated 24,627,178 raw reads from fecal samples collected across all time points. The sequencing depth per sample was adequate, with a mean of 139,803 reads, a median of 152,468 reads, and a maximum of 207,217 reads. The distribution of reads was comparable between experimental groups, with 12,222,322 reads derived from control animals and 12,079,860 reads from the CCFA-treated group. The quality of sequencing data were consistently high, as indicated by an average Phred quality score of 36.7. In addition, less than 1% of total reads had a Phred score below 22, which confirms the inclusion of minimal low-quality sequence reads and supports the reliability of downstream analyses. Taxonomic classification was performed using the MG-RAST^®^ platform, enabling the annotation of microbial communities across all samples. In total, 28 bacterial phyla and 587 genera were identified, underscoring the microbial diversity present in the gastrointestinal tract of neonatal lambs during the first two months of life.

### 2.3. Effects of the Early Ceftiofur Treatment on the Lamb Fecal Microbiome Composition and Diversity

**Figure 1:**
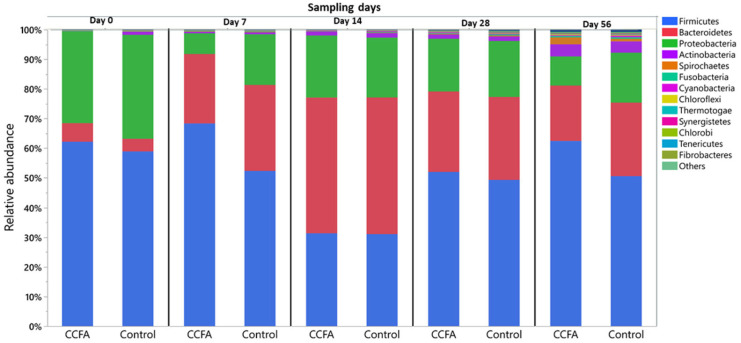
Taxonomic classification at the phylum level for the control and Ceftiofur (CCFA)-treated lambs at each sampling time point (day 0, day 7, day 14, day 28, and day 56). Only genera with ≥ 0.25% mean relative abundance are shown. Dominant genera included Bacteroides, Eubacterium, Streptococcus, Escherichia, and Clostridium.

Generally, the configuration and diversity of the fecal microbiota at both the phylum and genus levels varied between different time points in both the control and CCFA groups. At the phylum level, *Firmicutes* (52%), *Bacteroidetes* (25%), and *Proteobacteria* (20%) were the most predominant phyla in both the control and CCFA groups (Figure 1). The relative abundance of *Firmicutes* on day 0 represented 59% and 63% of all microbial populations in the control and CCFA-treated groups, respectively. On day 14, the proportion of Firmicutes decreased to half in favor of increased Bacteroidetes and Proteobacteria in both groups. On day 56, the *Firmicutes* proportions were restored and became the most abundant phylum, at 50% of the total in the control and 63% in the CCFA-treated group. When comparing the two groups, this increase in Firmicutes on day 56 was only significant (*p*-value = 0.015) in the CCFA group. Although the other two major phyla showed no significant change between groups at different time points, some minor phyla showed a substantial difference between the control and CCFA groups. These included *Actinobacteria* (*p*-value = 0.0008) at day 0, *Verrucomicrobia* (*p*-value = 0.046) at both day 0 and 56, *Fusobacteria* (*p*-value = 0.035) at day 14, and *Chlorobi* (*p*-value = 0.035) at day 28. The other bacterial phyla that averaged less than 0.25% of the total are described in Table S1.

**Figure 2:**
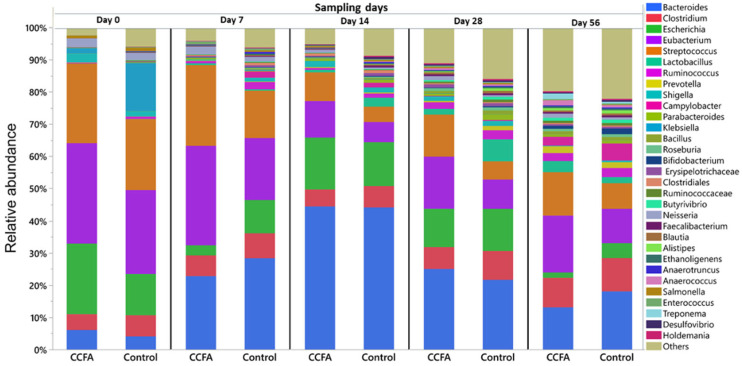
Taxonomic classification of WGSS at the genus level for the control and Ceftiofur (CCFA)-treated lambs at each sampling time (day 0, day 7, day 14, day 28, and day 56). Only those bacterial genera averaged more than 0.25% of the relative abundance across all samples are displayed.

At the genus level, the prevalent bacterial genera that averaged more than 0.25% of the total across all samples were *Bacteroides* (22.75%), *Eubacterium* (17.88%), *Streptococcus* (14.04%), *Escherichia* (10.93%), *Clostridium* (7.28%), *Lactobacillus* (1.86%), *Klebsiella* (1.76%), and *Ruminococcus* (1.59%). The distribution of the most abundant bacterial genera in both control and CCFA groups at different time points is illustrated in Figure 2. At all time points, there was a statistically significant decrease in the abundance of specific taxa in the CCFA-treated group compared to the control group. While there were taxa differences between groups at day 0 (*Campylobacter* (*p*-value = 0.049), *Klebsiella* (*p*-value = 0.045), and low abundance genera (*p*-value = 0.0117)), the number and identity of the taxa differences increased over time, from three (*Klebsiella* (*p*-value = 0.027), *Clostridiales* (*p*-value = 0.046), and *Salmonella* (*p*-value = 0.036)) on day 7 to six (*Ruminococcus (p*-value = 0.046), *Bacillus* (*p*-value = 0.0357), *Erysipelotrichaceae* (*p*-value = 0.0033), *Alistipes* (*p*-value = 0.0404), *Treponema* (*p*-value = 0.0156), and *Holdemania* (*p*-value = 0.0209)) on day 14. On day 28, only *Parabacteroides* showed a minimal difference between groups (p = 0.046), while no significant differences were observed on day 56. The other bacterial genera that averaged less than 0.25% are summarized in Table S2.

**Figure 3:**
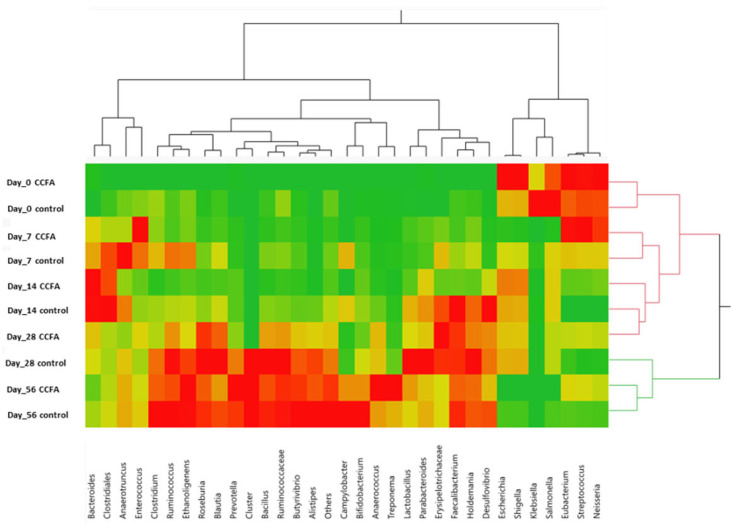
A heat map shows the predominant genera’s two-way clustering between the control and the CCFA group at different time points. Clustering analysis revealed similar microbial community structures between groups at days 0 to 14. By day 28, divergence became evident, indicating treatment-associated shifts. Partial convergence by day 56 suggests microbial recovery following CCFA-induced disruption.

The two-way clustering analysis comparing the predominant genera between the control and the CCFA-treated groups at different time points revealed overlapping clades at days 0, 7, and 14. However, the clustering pattern diminished on day 28 but reappeared on day 56, as shown in the heatmap (Figure 3).

**Figure 4:**
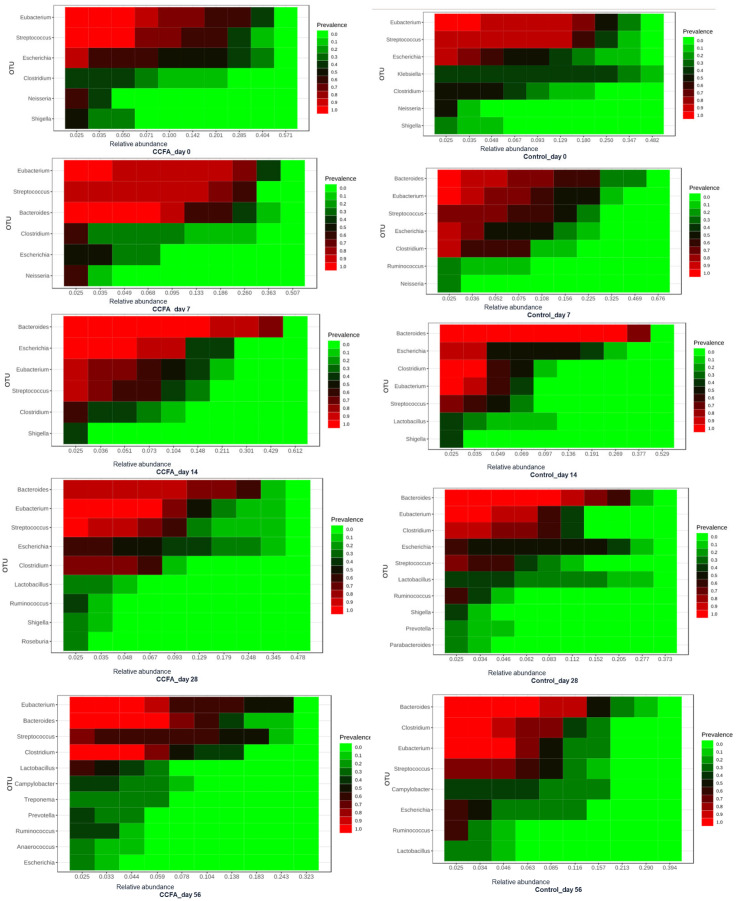
Heatmap of the relative abundance of core genera in CCFA-treated and control groups across time points. The heatmap displays the most abundant bacterial genera identified in fecal samples from control and CCFA-treated lambs at days 0, 7, 14, 28, and 56. A noticeable delay in the development of *Ruminococcus* and Lactobacillus was observed in the CCFA-treated group. By day 56, this group also showed unique enrichment of genera such as *Prevotella*, *Treponema*, and *Anaerococcus*, indicating altered microbial succession following antibiotic exposure.

The core microbiome heatmap showed the relative abundance of the richest genera in both control and CCFA-treated groups at each time point (Figure 4). It showed a clearer delay in *Ruminococcus* and *Lactobacillus* development in the CCFA-treated group than in the control group. In addition, the CCFA-treated group’s core microbiota encountered an exclusive enrichment of some genera, such as *Prevotella*, *Treponema*, and *Anaerococcus*, on day 56.

**Figure 5:**
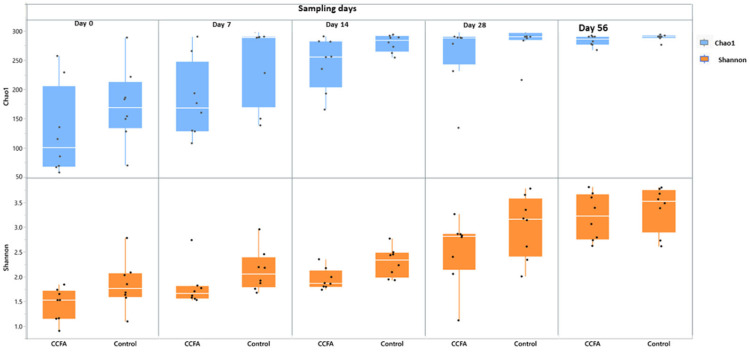
Alpha diversity of fecal microbiota in CCFA-treated and control lambs across sampling time points, assessed using Chao1 and Shannon indices. Both richness matrices increased significantly over time in both groups. However, the Shannon index showed reduced genus-level diversity in the CCFA group on days 7 and 14.

**Figure 6:**
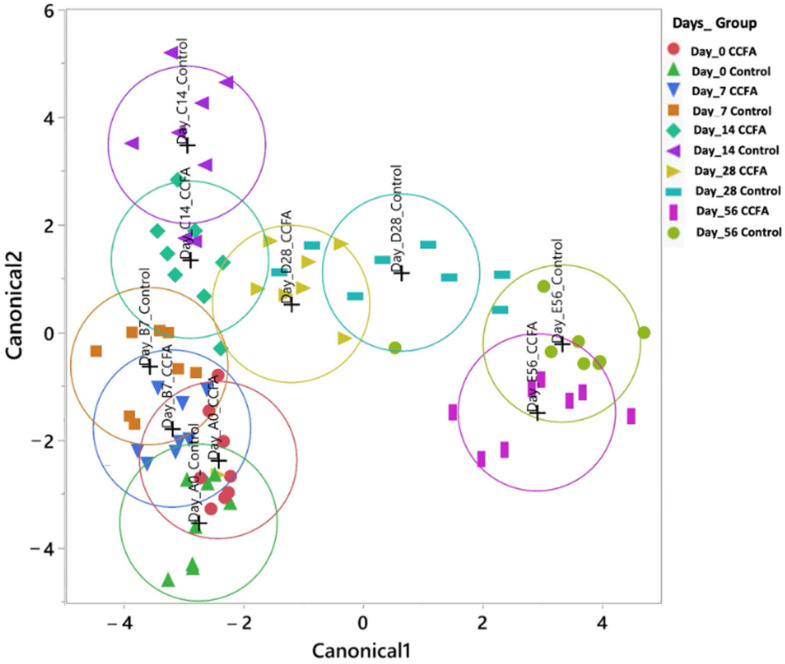
Canonical discriminant analysis of beta diversity in fecal microbiota across time in CCFA-treated and control lambs. A significant shift in beta diversity was observed over time, with distinct clustering between CCFA-treated and control groups at each time point.

The fecal microbiota alpha-diversity for the predominant genera was evaluated using Chao1 and Shannon metrics and the non-parametric tests. Both metrics showed a significant increase in diversity and richness (*p*-value = 0.001) over time in both groups. However, the Shannon index revealed that the genus richness was significantly less in the CCFA-treated group than in the control group on day 7 (*p*-value = 0.03) and day 14 (*p*-value = 0.01) (Figure 5). Discriminant analysis showed a significant (*p*-value = 0.001) and similar change in beta diversity in both the CCFA and control groups over time, but with an apparent pattern of clustering between groups at each time point (Figure 6).

### 2.4. Normal Fecal Microbiota Development

**Figure 7:**
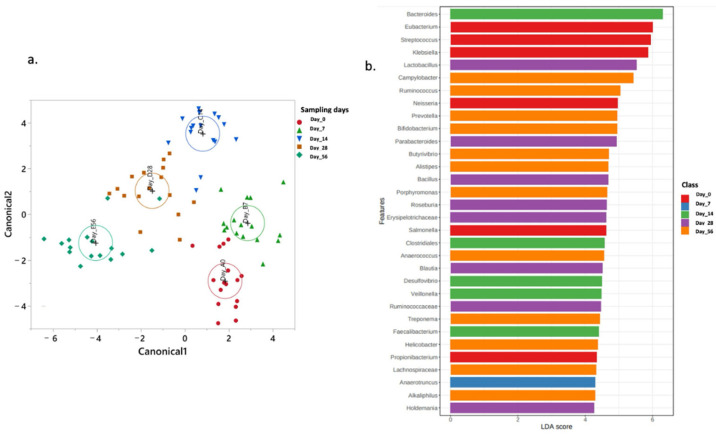
(**a**) Canonical discriminant analysis (CDA) showing temporal clustering of genera in the control group from day 0 to day 56. (**b**) Linear discriminant analysis (LEfSe) of genera with > 0.25% mean relative abundance across time points, highlighting stage-specific microbial enrichment.

The normal lamb fecal microbiota development over time was depicted in the control group using canonical discriminant analysis (CDA), which showed the overall microbial population at the different time points of the experiment (Figure 7a). Using the LEfSe algorithm, the most enriched genera with relative abundance averaging more than 0.25 were *Eubacterium, Streptococcus, Klebsiella, Neisseria, Salmonella, Propionibacterium, Anaerotruncus, Bacteroidetes, Colstridiales, Desulfovibrio, Veillonella*, and *Faecalibacterium* in the nursing stage (Figure 7b). The most enriched genera during the early phase after weaning comprise *Lactobacillus, Parabacteroides, Bacillus, Rosiburia, Erysipelotrichaceae, Blautia, Ruminococcaceae, Holdemania, Campylobacter, Rumicoccus, Prevotella, Bifidobacterium, Butyrivibrio, Alistipes, Prophyromonas, Anaerococcus, Treponema, Helicobacter, Lachnospiraceae*, and *Alkaliphilus*.

### 2.5. Effect of Ceftiofur on the Microbial Functional Resistance Genes

**Figure 8:**
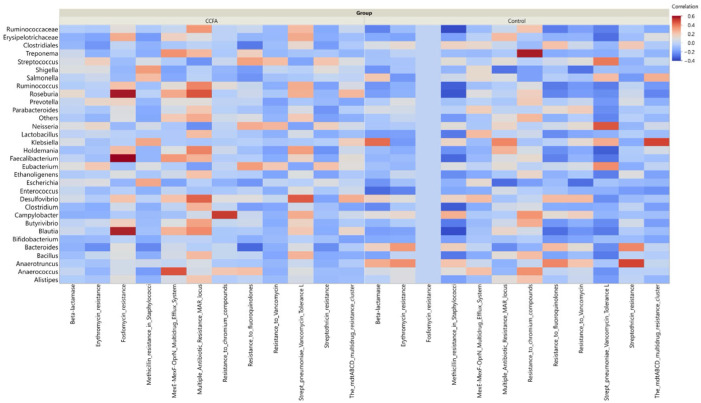
Correlation heatmap between enriched bacterial genera and predicted antimicrobial resistance genes (ARGs) in CCFA-treated and control lambs. The CCFA group showed stronger correlations between core taxa and ARGs.

**Figure 9:**
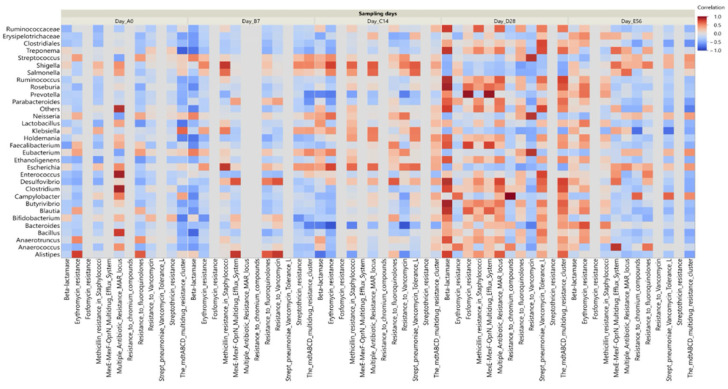
Correlation heatmap between enriched genera and functional ARGs in the CCFA-treated group across all sampling time points. Enrichment and correlation of resistance genes were most evident on day 28, particularly with beta-lactam and other multidrug resistance.

The annotated functional profile reads that the correlation between the number of potential antibiotic-resistant genes and the relative abundance of some core microbiome taxa was higher in the CCFA group than in the control group (Figure 8). We investigated this correlation in the CCFA group at different time points (Figure 9). Beta-lactamase-resistant genes had the highest abundance in the CCFA group, especially on day 28. The number of resistance genes was significantly correlated with the relative abundance of *Bacteroides, Bacillus, Ruminococcus, Roseburia, Treponema, Butyrivibrio, Desulfovibrio, Faecalibacterium, Anaerotruncus*, and the low-abundance genera (*p*-value ≤ 0.05). In addition, there was a significant increase in the relative abundance of other antibiotic-resistant genes with specific taxa at different time points, and they were more concentrated on day 28 (Figure 9).

## 3. Discussion

This study used WGSS to analyze early gut microbiome development in lambs and the microbial community perturbations after a single intramuscular injection of CCFA. We also examined the potential emergence of antimicrobial resistance genes in these early-life microbial communities. The initial fecal microbial populations comprised three main phyla: *Firmicutes, Bacteroidetes,* and *Proteobacteria,* similar to those described in the developing gastrointestinal tract of other monogastric and herbivorous animals [26]. These taxa are present in colostrum and are consistent with the notion that colostrum contributes to seeding such microbial communities in the neonatal intestine [27]. Our study’s apparent variation in the relative abundance of these three taxa over time aligns with the results reported in Calves [7]. The other prevalent bacteria were *Bacteroides, Eubacterium, Streptococcus, Escherichia, Clostridium, Lactobacillus, Klebsiella*, and *Ruminococcus.* Each genus has been shown to create anaerobic conditions essential for the growth of other beneficial anaerobes in the ruminant gastrointestinal tract [28,29]. Our data showed a progressive and highly significant progression of diversity and richness in these taxa, with the increasing age of the lambs strongly indicating these organisms’ importance in the temporal development of the alimentary system [30].

One unique aspect of this study was evaluating microbial changes with lamb age over two stages of husbandry and development: nursing and grazing. The presence of specific microbial taxa during these health management stages is critical for the maturation and regulation of various physio-immunological processes [31,32]. In addition, a dysbiosis of these pioneer taxa has been associated with gastrointestinal diseases, growth disruption, and potential immune dysregulation [8,33]. Notably, several of the predominant taxa observed in our experimental lambs have been shown to be affected by nutrition and maturity and are associated with immune status and health outcomes in other ruminant species. For instance, a high abundance of *Prevotella* and *Ruminococcus* has been linked to forage intake [30,34]. In pre-weaned calves, the increased relative abundance of *Prevotella* was positively correlated with better growth performance [35]. Lastly, high proportions of *Propionibacterium* and *Faecalibacterium* taxa have been linked to immune regulation, increased weight gain, and a reduction in the incidence of diarrhea in young calves [8].

Another key finding in this study was the transient impact of a single injection of CCFA on the microbiota of the neonatal lamb gastrointestinal tract. Ceftiofur (CCFA) was selected as the antibiotic of interest in our study because of its widespread use in the livestock industry and its importance as a human therapeutic [36,37,38]. It is a third-generation cephalosporin with an extended duration of action, effective against a wide range of Gram-positive and Gram-negative bacteria. Although the main route of its administration is parenteral, its active derivatives are partially eliminated via bile into the intestinal tract, where they can influence gut microbial populations. This biliary excretion enables CCFA to impact intestinal microbiota despite its systemic administration [22]. Sensitive microbial taxa, particularly those involved in early colonization, may be transiently suppressed. Moreover, the low but sustained presence of the drug due to enterohepatic circulation can prolong its effects in the intestinal lumen and potentially promote conditions that favor the survival of resistant organisms [39]. The longevity of low-level exposure of the gastrointestinal microbial community to CCFA following a single parenteral injection matches the temporal pattern of change observed in the treated lambs in our study.

An essential aspect of the design of this study was to ensure that any observed differences in microbial community composition between experimental groups were due to the treatment effect and not a result of individual variation. The presence of a confounding difference in pre-treatment and the initial differences in the microbial relative abundances between the CCFA-treated and the control groups at day 0 within a few phyla or genera did not significantly affect or even become a permanent signature at subsequent time points. These slight differences in microbiome composition at this early age could be attributed to variations in maternal influence or could result from secondary influences from factors such as colostral intake [15,40].

In our study, differences in the configuration and diversity of the fecal microbiota between experimental groups were observed at both the phylum and the genus level. The extent and nature of these changes varied over time, but after two months, there was almost no significant difference between control and antimicrobial-treated groups. These findings show both concordance and discordance with existing literature in some phyla and/or taxa; for details, see (Table S3). The overall trajectory of CCFA-associated change was a decrease in abundance (*Salmonella* and unclassified derived *Clostridiales; Bacillus, Ruminococcus*, *Holdemania*, and Alistipes). Firmicutes were the only taxa with a significant increase, and this change was only observable two months after the antimicrobial exposure. The abundance of this taxon has been correlated with health and growth in growing pigs [41]. Observing short-term changes in *Salmonella,* unclassified derived *Clostridiales*, and *Bacillus* is compatible with their in vitro sensitivity to ceftiofur [42]. Public health concerns exist regarding the former two taxa since they can degrade and build resistance to cephalosporins [43]. Early life and short-term declines in the *Ruminococcus*, *Holdemania*, and *Alistipes* taxa are unlikely to be biologically significant since the lambs are still in the nursing stage and at an early stage of gastrointestinal development. These taxa are involved in cellulose digestion and rumen development and are essential for rumen development in the grazing period [32,44].

Further evaluation of the fecal microbial diversity between the CCFA-treated and control lambs revealed apparent microbial community changes with potential antibiotic resistance development. Although previous studies showed a significant decrease in alpha and beta diversity after ceftiofur treatment [45], our study revealed insignificant differences between the two groups using the Bray-Curtis beta diversity index. However, there was a tendency (*p*-value = 0.14) for a complete microbial dissimilarity between the two groups on day 14. This pattern effect of the CCFA over time was also apparent in the two-way clustering comparison between the CCFA and the control groups. Additionally, assessment of the core microbiome between the CCFA-treated and control groups at each time point showed a delay in the development of *Ruminococcus* and *Lactobacillus* in the CCFA group, which might have a temporary adverse effect on the lamb’s health [2]. Moreover, on day 28 and day 56, the CCFA-treated lambs showed enrichment of *Roseburia*, *Treponema*, and *Anaerococcus*. Although they were members of the normal microbiome development in the control lambs, the LDA revealed their enrichment in the CCFA-treated lambs, which may be associated with these organisms’ rapid adaptation and antibiotic resistance development [46]. The low exposure of the gastrointestinal microbial community to CCFA raises concern regarding the possible emergence of antimicrobial resistance following a single injection [37].

The most crucial finding in this study was that the CCFA-associated changes were relatively small in scope and of short duration. While it has been established that the reduction in microbial taxa in the early developmental stages might leave a permanent change in the adulthood microbial communities with an increased risk of reduced production or immunological diseases [47], there is growing evidence that host genetics can homeostatically modulate this disturbance with increasing age [48,49,50], besides the recent report by Liu et al. (2022), who described that the gut microbiome could develop resilience to antibiotic perturbation [51]. This might explain the minimal differences between the CCFA-treated lambs and the control group on day 28 and the non-significant changes on day 56. Overall, these findings highlight the complex interplay between antibiotic treatment and microbial community dynamics, underscoring the importance of considering both short- and long-term effects when assessing the impact of antimicrobial interventions on gut microbiota.

Unlike the transient effect of the CCFA on the differences in the microbial relative abundances and diversities between the CCFA-treated group and the control group, the development of antibiotic resistance, including both specific and multidrug-resistant genes, can persist for a long time. This persistence is due to the horizontal transfer of these genes within and between hosts and into their environment, resulting in a significant concern for both animal and human health [52,53,54]. In this study, the correlation between the relative abundance of potential resistant genes from the functional analysis and the relative abundance of prevalent taxa was most pronounced in ceftiofur-resistant bacteria. This correlation was notable on day 14 and became more pronounced by day 28, likely due to the time required for bacteria to express resistant genes. For instance, it has been shown that it takes about 17 days for calves exposed to a therapeutic dose of ceftiofur to express these genes [55,56].

In contrast to the findings of Weinroth et al. (2018), who reported no association of ceftiofur treatment with beta-lactamase resistance in cattle [57], our results showed that the beta-lactam-resistant genes were evident for most of the prevalent taxa. This observation aligns with the findings by Dong et al. (2022) [58]. Moreover, ceftiofur-resistant genes are known to be transferable within and between bacterial genera, which could explain their spread among the predominant genera in our study [56]. Consistent with previous research, our results indicate that the presence of potentially resistant genes varies over time [36]. Lastly, the widespread use of ceftiofur and its impact on the gut resistome make it a pertinent focus for understanding the long-term implications of antibiotic practices on animal and human health. We anticipate that specific and multidrug-resistant genes within the enriched gut antibiotic resistome could persist for extended periods, facilitated by horizontal gene transfer to other hosts and their environment [54,59]. These findings highlight the critical need for ongoing monitoring and management of antibiotic practices to mitigate the long-term impacts of antibiotic use on both animal and human health.

## 4. Materials and Methods

### 4.1. Experimental Design and Sample Collection

#### 4.1.1. Animals

A total of 24 healthy neonatal lambs were included in this study, assigned into two groups: CCFA-treated (n = 12) and control (n = 12). Within the CCFA-treated group were five males and seven females, while the control group had four males and eight females. The average body weight (kg) of the CCFA-treated and the control groups was 4.1 ± 0.62 and 3.8 ± 0.88, respectively.

#### 4.1.2. Challenge

The treated lambs have received a single intramuscular dose of ceftiofur crystalline free acid (CCFA^a^, Excede^®^, Zoetis Inc., Parsippany, NJ, USA) at 5.0 mg/kg 24 hrs after birth [60].

#### 4.1.3. Samples

Deep fecal swabs from both groups were collected at five time points representing two lambs’ developmental stages: the nursing stage that represents day 0 (D0), day 7 (D7), and day 14 (D14), and the start of the grazing stage that comprises day 28 (D28) and day 56 (D56).

#### 4.1.4. Ethics Statement of Animal Use

All procedures in this study were conducted in the university research sheep farm under the agreement and protocol guidelines of the Institutional Animal Care and Use Committee (IACUC) at the University of Illinois, Urbana-Champaign. 

### 4.2. DNA Extraction

According to the manufacturer’s instructions, DNA was extracted from each sample using the QIAGEN QIAamp power fecal DNA extraction kit (Cat. No. 12830, MO BIO Laboratories, Inc., Carlsbad, CA, USA). The concentration and purity of the extracted DNA were evaluated using a NanoDrop™ spectrophotometer (NanoDrop Technologies, Rockland, DE, USA) at wavelengths of 260 and 280 nm. The DNA integrity was assessed by running gel electrophoresis (Bio-Rad Laboratories, Inc., Hercules, CA, USA) and stored at −20 °C until sequencing.

### 4.3. Whole Genome Shotgun DNA Sequencing (WGSS) 

The extracted DNA was sent on dry ice to the W. M. Keck Center for Comparative and Functional Genomics (the University of Illinois at Urbana-Champaign, Urbana, IL, USA), where the WGSS workflow was carried out. The initial step started with the Nextera DNA Flex Library Preparation Kit, which was used to build the DNA libraries (Illumina, Inc., San Diego, CA, USA). Briefly, aliquots of 100 ng of DNA were cleaned by magnetic beads and amplified five times via Illumina Enhanced PCR Mix and Nextera FS dual-indexed primers. The amplified DNAs were subjected to another cleaning process, and fragments between 250 and 750 bp were purified using a double-sided bead purification procedure. The final products were measured using the Qubit DNA quantifier (Life Technologies, Grand Island, NY, USA) [61]. The AATI Fragment Analyzer (Advanced Analytics, Ames, IA, USA) detected the mean size. After uniform amalgamation of the libraries, they were cleaned using a 1:1 ratio with AxyPrep Mag PCR Cleanup beads (Axygen, Inc., Union City, CA, USA) and checked for size for a second time using an AATI Fragment Analyzer. Then, only 5 nM of the final pool was further quantified using qPCR (Bio-Rad Laboratories, Inc., CA, USA) and exposed for denaturation, and then a 4% non-indexed PhiX control library was loaded at a concentration of 10 pM into the MiSeq V3 flowcell for cluster formation and sequencing. Finally, DNA sequencing proceeded from both ends of the molecules, forming reads of 250 nt from each end following the manufacturer’s guidelines (Illumina, Inc., San Diego, CA, USA).

### 4.4. Bioinformatic Analysis

#### 4.4.1. Sequence Reads Processing

The raw sequence reads were demultiplexed and transformed into Fastq files using Casava v.1.8.2 (Illumina, Inc., San Diego, CA, USA), then evaluated by the FastQC software (version 0.12.1; Babraham Bioinformatics, Cambridge, UK) [62]. After that, Trimmomatic software (version 0.39; Bolger et al., 2014) [63] was used to snip low-resolution reads (<30 on Phred score) and the adaptor sequences. The sequence files were synced via the Subsystems Technology (MG-RAST, Lemont, IL, USA) web server into the Metagenome Rapid Annotation, where the microbial composition was ready to be analyzed [64]. This web server involves extra refinement processes for the reads, such as length filtering and ambiguous base filtering. These processes ensure retaining all sequences with a standard deviation ≤ 2 and those containing less than five dubious base pairs. In addition, the reads were fine-tuned by removing both host-exclusive species read sequences as well as other sequencing-based artifact reads. Eventually, the filtered sequences were subjected to normalization and standardization processes. Normalization was carried out by log_2_-based transformation [log_2_(x + 1)], and the standardization process was constructed within each sample and linearly between all samples.

#### 4.4.2. Operational Taxonomic Units (OTUs) Analysis

The MG-RAST web server analyzed the OTUs of the fecal microbiota at the phylum, genus, and species levels and metabolic functional gene profiles [65]. It identifies and converts millions of metagenomic sequence reads into discrete microorganisms using the rapid and highly efficient inquisitive algorithms within the genome databases. The microbial classification was accomplished by a nonredundant multisource protein annotation database (M5NR). The relative abundance was analyzed via the best-hit approach with a maximum e value of 1 × 10^−5^, a minimum identity cutoff of 60%, and a minimum alignment length cutoff of 15 [66]. The microbiome analyst computed both alpha and beta diversity indices of the fecal microbiota. Chao 1 and Shannon indices were used to demonstrate alpha diversity. In contrast, beta diversity was determined by principal component analysis (PCA) based on a non-phylogenetic built-in Bray-Curtis distance metric [67]. The predicted functional gene profiles were elucidated using the SEED annotation system. All sequence reads could be accessed through the NCBI with the Bioproject ID number PRJNA917185.

#### 4.4.3. Beta-Lactam Resistome Identification

The predicted antibiotic-resistant genes (ARGs) to ceftiofur and the relative abundance of beta-lactam-resistant genes and other resistant genes in the functional profile were calculated using MG-RAST and then correlated to the relative abundance of the taxa identified in the fecal microbiota using the JMP Pro 13^®^ software (SAS Institute Inc., Cary, NC, USA).

### 4.5. Statistical Analysis 

The difference in the overall microbial composition and beta diversity between the control and the CCFA-treated groups was assessed using the non-parametric multivariate analysis of variance (PERMANOVA) with 9999 permutations and Bonferroni-corrected *p*-values using the microbiome analyst^®^online platform at each time point (D0, D7, D14, D28, and D56). The difference in fecal microbiota relative abundance and alpha diversity between the two groups was estimated by the Mann–Whitney pairwise comparison test with sequential Bonferroni significance at a *p*-value < 0.05 using the JMP Pro 13^®^ software (SAS Institute Inc., Cary, NC, USA). The overall difference in the relative abundance and diversity of the ARGs between the control and CCFA-treated groups was computed using the same statistical method. The similarities within the overall microbial composition between the two groups at each time point were additionally assessed using linear discriminant analysis effect size (LEfSe) via Galaxy1^®^ [68]. On the other hand, to evaluate the overall predictive function of gene profiles between the control and CCFA-treated groups, the differences were compared using principal component analysis (PCA) and heatmaps using the JMP Pro 13^®^ software (SAS Institute Inc., Cary, NC, USA).

## 5. Conclusions

This study demonstrated sheep’s normal early gut microbiome development and ability to withstand antibiotic perturbation over time. A single intramuscular dose of ceftiofur crystalline-free acid in neonatal lambs can cause transitory disturbances to the average microbial profile, posing potential risks to the animal’s general health. These risks arise from reducing specific important taxa during a critical developmental stage, alongside the likely long-term emergence of antimicrobial resistance.

## Data Availability

The data are available at the NCBI with the BioProject ID number PRJNA917185.

## Supplementary Materials

The following supporting information can be downloaded at https://www.mdpi.com/article/10.3390/antibiotics14050434/s1, Table S1: taxonomic classification of the fecal microbiome at the phylum level for both control and Ceftiofur (CCFA) treated lambs at each sampling time point (day 0, day 7, day 14, day 28, and day 56). This table summarizes bacterial phyla of relative abundance averaged more than 0.25% across all samples. Table S2: taxonomic classification of the fecal microbiome at the phylum level for both control and Ceftiofur (CCFA) treated lambs at each sampling time point (day 0, day 7, day 14, day 28, and day 56). All other bacterial phyla of relative abundance averaged less than 0.25% across all samples are included in this table: Table S3: summary table of the significant microbiome changes in CCFA-treated lambs over time with supporting evidence from the literature. References [22,46,69,70,71,72] are cited in the Supplementary Materials.

## Abbreviations

The following abbreviations are used in this manuscript:

CCFA	Ceftiofur Crystalline Free Acid
ARG(s)	Antibiotic Resistance Gene(s)
WGSS	Whole Genome Shotgun Sequencing
DNA	Deoxyribonucleic Acid
OTUs	Operational Taxonomic Units
MG-RAST	Metagenomics Rapid Annotation using Subsystem Technology
RefSeq	Reference Sequence Database
PCA	Principal Component Analysis
CDA	Canonical Discriminant Analysis
LDA	Linear Discriminant Analysis
LEfSe	Linear Discriminant Analysis Effect Size
PCR	Polymerase Chain Reaction

## References

[1] Alonso V.R., Guarner F. (2013). Linking the gut microbiota to human health. Br. J. Nutr..

[2] Malmuthuge N., Griebel P.J., Guan L.L. (2015). The gut microbiome and its potential role in the development and function of newborn calf gastrointestinal tract. Front. Vet. Sci..

[3] Van Den Broek M.F., De Boeck I., Kiekens F., Boudewyns A., Vanderveken O.M., Lebeer S. (2019). Translating recent microbiome insights in otitis media into probiotic strategies. Clin. Microbiol. Rev..

[4] Khalil A., Batool A., Arif S. (2022). Healthy Cattle Microbiome and Dysbiosis in Diseased Phenotypes. Ruminants.

[5] Kraimi N., Dawkins M., Gebhardt-Henrich S.G., Velge P., Rychlik I., Volf J., Creach P., Smith A., Colles F., Leterrier C. (2019). Influence of the microbiota-gut-brain axis on behavior and welfare in farm animals: A review. Physiol. Behav..

[6] Guo C.Y., Ji S.K., Yan H., Wang Y.J., Liu J.J., Cao Z.J., Yang H.J., Zhang W.J., Li S.L. (2020). Dynamic change of the gastrointestinal bacterial ecology in cows from birth to adulthood. MicrobiologyOpen.

[7] Li R.W., Connor E.E., Li C., Baldwin V., Ransom L., Sparks M.E. (2012). Characterization of the rumen microbiota of pre-ruminant calves using metagenomic tools. Environ. Microbiol..

[8] Oikonomou G., Teixeira A.G.V., Foditsch C., Bicalho M.L., Machado V.S., Bicalho R.C. (2013). Fecal microbial diversity in pre-weaned dairy calves as described by pyrosequencing of metagenomic 16S rDNA. Associations of Faecalibacterium species with health and growth. PLoS ONE.

[9] Malmuthuge N., Griebel P.J., Guan L.L. (2014). Taxonomic identification of commensal bacteria associated with the mucosa and digesta throughout the gastrointestinal tracts of preweaned calves. Appl. Environ. Microbiol..

[10] Malmuthuge N., Li M., Chen Y., Fries P., Griebel P.J., Baurhoo B., Zhao X., Guan L.L. (2012). Distinct commensal bacteria associated with ingesta and mucosal epithelium in the gastrointestinal tracts of calves and chickens. FEMS Microbiol. Ecol..

[11] Sommer F., Bäckhed F. (2013). The gut microbiota—Masters of host development and physiology. Nat. Rev. Microbiol..

[12] Henderson G., Cox F., Ganesh S., Jonker A., Young W., Janssen P.H. (2015). Rumen microbial community composition varies with diet and host, but a core microbiome is found across a wide geographical range. Sci. Rep..

[13] Weimer P., Stevenson D., Mantovani H., Man S. (2010). Host specificity of the ruminal bacterial community in the dairy cow following near-total exchange of ruminal contents. J. Dairy Sci..

[14] Benson A.K., Kelly S.A., Legge R., Ma F., Low S.J., Kim J., Zhang M., Oh P.L., Nehrenberg D., Hua K. (2010). Individuality in gut microbiota composition is a complex polygenic trait shaped by multiple environmental and host genetic factors. Proc. Natl. Acad. Sci. USA.

[15] Yeoman C.J., Ishaq S.L., Bichi E., Olivo S.K., Lowe J., Aldridge B.M. (2018). Biogeographical differences in the influence of maternal microbial sources on the early successional development of the bovine neonatal gastrointestinal tract. Sci. Rep..

[16] Cammack K.M., Austin K.J., Lamberson W.R., Conant G.C., Cunningham H.C. (2018). Ruminnat nutrition symposium: Tiny but mighty: The role of the rumen microbes in livestock production. J. Anim. Sci..

[17] Wang J., Fan H., Han Y., Zhao J., Zhou Z. (2016). Characterization of the microbial communities along the gastrointestinal tract of sheep by 454 pyrosequencing analysis. Asian-Australas. J. Anim. Sci..

[18] Li K., Shi B., Na R. (2023). The colonization of rumen microbiota and intervention in pre-weaned ruminants. Animals.

[19] Xiao H., Yan H., Tian P., Ji S., Zhao W., Lu C., Zhang Y., Liu Y. (2023). The effect of early colonized gut microbiota on the growth performance of suckling lambs. Front. Microbiol..

[20] Blaser M.J. (2016). Antibiotic use and its consequences for the normal microbiome. Science.

[21] Batey R., Nilon P., Page S., Browning G., Norris J. (2024). Antimicrobial prescribing guidelines for sheep. Aust. Vet. J..

[22] Foster D.M., Jacob M.E., Farmer K.A., Callahan B.J., Theriot C.M., Kathariou S., Cernicchiaro N., Prange T., Papich M.G. (2019). Ceftiofur formulation differentially affects the intestinal drug concentration, resistance of fecal *Escherichia coli*, and the microbiome of steers. PLoS ONE.

[23] Rivera-Garcia S., Angelos J.A., Rowe J.D., Byrne B.A., Wetzlich S.E., Van Liew D.B., Tell L.A. (2014). Pharmacokinetics of ceftiofur crystalline-free acid following subcutaneous administration of a single dose to sheep. Am. J. Vet. Res..

[24] Sawant A., Sordillo L., Jayarao B. (2005). A survey on antibiotic usage in dairy herds in Pennsylvania. J. Dairy Sci..

[25] Wang C., Wei S., Chen N., Xiang Y., Wang Y., Jin M. (2022). Characteristics of gut microbiota in pigs with different breeds, growth periods and genders. Microb. Biotechnol..

[26] Kobayashi R., Nagaoka K., Nishimura N., Koike S., Takahashi E., Niimi K., Murase H., Kinjo T., Tsukahara T., Inoue R. (2020). Comparison of the fecal microbiota of two monogastric herbivorous and five omnivorous mammals. Anim. Sci. J..

[27] Esteban-Blanco C., Gutiérrez-Gil B., Marina H., Pelayo R., Suárez-Vega A., Acedo A., Arranz J.-J. (2020). The milk microbiota of the spanish churra sheep breed: New insights into the complexity of the milk microbiome of dairy species. Animals.

[28] Pantoja-Feliciano I.G., Clemente J.C., Costello E.K., Perez M.E., Blaser M.J., Knight R., Dominguez-Bello M.G. (2013). Biphasic assembly of the murine intestinal microbiota during early development. ISME J..

[29] Minato H., Otsuka M., Shirasaka S., Itabashi H., Mitsumori M. (1992). Colonization of microorganisms in the rumen of young calves. J. Gen. Appl. Microbiol..

[30] Jami E., Israel A., Kotser A., Mizrahi I. (2013). Exploring the bovine rumen bacterial community from birth to adulthood. ISME J..

[31] Nicholson J.K., Holmes E., Kinross J., Burcelin R., Gibson G., Jia W., Pettersson S. (2012). Host-gut microbiota metabolic interactions. Science.

[32] Arshad M.A., Hassan F.-U., Rehman M.S., Huws S.A., Cheng Y., Din A.U. (2021). Gut microbiome colonization and development in neonatal ruminants: Strategies, prospects, and opportunities. Anim. Nutr..

[33] O’Mahony L., McCarthy J., Kelly P., Hurley G., Luo F., Chen K., O’Sullivan G.C., Kiely B., Collins J.K., Shanahan F. (2005). Lactobacillus and bifidobacterium in irritable bowel syndrome: Symptom responses and relationship to cytokine profiles. Gastroenterology.

[34] Kim Y.-H., Nagata R., Ohtani N., Ichijo T., Ikuta K., Sato S. (2016). Effects of dietary forage and calf starter diet on ruminal pH and bacteria in Holstein calves during weaning transition. Front. Microbiol..

[35] Kim E.-T., Lee S.-J., Kim T.-Y., Lee H.-G., Atikur R.M., Gu B.-H., Kim D.-H., Park B.-Y., Son J.-K., Kim M.-H. (2021). Dynamic changes in fecal microbial communities of neonatal dairy calves by aging and diarrhea. Animals.

[36] Singer R.S., Patterson S.K., Wallace R.L. (2008). Effects of therapeutic ceftiofur administration to dairy cattle on *Escherichia coli* dynamics in the intestinal tract. Appl. Environ. Microbiol..

[37] Hornish R.E., Katarski S. (2002). Cephalosporins in veterinary medicine-ceftiofur use in food animals. Curr. Top. Med. Chem..

[38] Wren C.M., Cowper J., Greer N., Goldin L., Perry A. (2022). Effect of reduced fluoroquinolone use on cephalosporin use, susceptibilities and Clostridioides difficile infections. Antibiotics.

[39] Beyer A., Baumann S., Scherz G., Stahl J., von Bergen M., Friese A., Roesler U., Kietzmann M., Honscha W. (2015). Effects of ceftiofur treatment on the susceptibility of commensal porcine E. coli–comparison between treated and untreated animals housed in the same stable. BMC Vet. Res..

[40] Song Y., Li F., Fischer-Tlustos A., Neves A., He Z., Steele M., Guan L. (2021). Metagenomic analysis revealed the individualized shift in ileal microbiome of neonatal calves in response to delaying the first colostrum feeding. J. Dairy Sci..

[41] Ruczizka U., Metzler-Zebeli B., Unterweger C., Mann E., Schwarz L., Knecht C., Hennig-Pauka I. (2019). Early parenteral administration of ceftiofur has gender-specific short-and long-term effects on the fecal microbiota and growth in pigs from the suckling to growing phase. Animals.

[42] Iwiński H., Wódz K., Chodkowska K., Nowak T., Różański H. (2022). In vitro evaluation of antimicrobial effect of phytobiotics mixture on Salmonella spp. isolated from chicken broiler. Antibiotics.

[43] VT Nair D., Venkitanarayanan K., Kollanoor Johny A. (2018). Antibiotic-resistant Salmonella in the food supply and the potential role of antibiotic alternatives for control. Foods.

[44] Deusch S., Tilocca B., Camarinha-Silva A., Seifert J. (2015). News in livestock research—Use of Omics-technologies to study the microbiota in the gastrointestinal tract of farm animals. Comput. Struct. Biotechnol. J..

[45] Nowland T.L., Torok V.A., Low W.Y., Barton M.D., Plush K.J., Kirkwood R.N. (2020). Faecal microbiota analysis of piglets during lactation. Animals.

[46] Rutjens S., Vereecke N., De Spiegelaere W., Croubels S., Devreese M. (2022). Intestinal Exposure to Ceftiofur and Cefquinome after Intramuscular Treatment and the Impact of Ceftiofur on the Pig Fecal Microbiome and Resistome. Antibiotics.

[47] Candon S., Perez-Arroyo A., Marquet C., Valette F., Foray A.-P., Pelletier B., Milani C., Ventura M., Bach J.-F., Chatenoud L. (2015). Antibiotics in early life alter the gut microbiome and increase disease incidence in a spontaneous mouse model of autoimmune insulin-dependent diabetes. PLoS ONE.

[48] Huda M.N., Salvador A.C., Barrington W.T., Gacasan C.A., D’Souza E.M., Ramirez L.D., Threadgill D.W., Bennett B.J. (2022). Gut microbiota and host genetics modulate the effect of diverse diet patterns on metabolic health. Front. Nutr..

[49] Thompson C.L., Wang B., Holmes A.J. (2008). The immediate environment during postnatal development has long-term impact on gut community structure in pigs. ISME J..

[50] Schwarzer M., Strigini M., Leulier F. (2018). Gut microbiota and host juvenile growth. Calcif. Tissue Int..

[51] Liu L., Kirst M.E., Zhao L., Li E., Wang G.P. (2022). Microbiome Resilience despite a Profound Loss of Minority Microbiota following Clindamycin Challenge in Humanized Gnotobiotic Mice. Microbiol. Spectr..

[52] Bengtsson-Palme J., Kristiansson E., Larsson D.J. (2018). Environmental factors influencing the development and spread of antibiotic resistance. FEMS Microbiol. Rev..

[53] Berglund B. (2015). Environmental dissemination of antibiotic resistance genes and correlation to anthropogenic contamination with antibiotics. Infect. Ecol. Epidemiol..

[54] Gasparrini A.J., Wang B., Sun X., Kennedy E.A., Hernandez-Leyva A., Ndao I.M., Tarr P.I., Warner B.B., Dantas G. (2019). Persistent metagenomic signatures of early-life hospitalization and antibiotic treatment in the infant gut microbiota and resistome. Nat. Microbiol..

[55] Sheedy D.B., Okello E., Williams D.R., Precht K., Cella E., Lehenbauer T.W., Aly S.S. (2021). Effect of antimicrobial treatment on the dynamics of ceftiofur resistance in Enterobacteriaceae from adult California dairy cows. Microorganisms.

[56] Jiang X., Yang H., Dettman B., Doyle M.P. (2006). Analysis of fecal microbial flora for antibiotic resistance in ceftiofur-treated calves. Foodbourne Pathog. Dis..

[57] Weinroth M.D., Scott H.M., Norby B., Loneragan G.H., Noyes N.R., Rovira P., Doster E., Yang X., Woerner D.R., Morley P.S. (2018). Effects of ceftiofur and chlortetracycline on the resistomes of feedlot cattle. Appl. Environ. Microbiol..

[58] Dong L., Meng L., Liu H., Wu H., Schroyen M., Zheng N., Wang J. (2022). Effect of Cephalosporin Treatment on the Microbiota and Antibiotic Resistance Genes in Feces of Dairy Cows with Clinical Mastitis. Antibiotics.

[59] Gibson M.K., Crofts T.S., Dantas G. (2015). Antibiotics and the developing infant gut microbiota and resistome. Curr. Opin. Microbiol..

[60] Martin K.L., Clapham M.O., Davis J.L., Baynes R.E., Lin Z., Vickroy T.W., Riviere J.E., Tell L.A. (2018). Extralabel drug use in small ruminants. J. Am. Vet. Med. Assoc..

[61] McMahon T., Abdelmesih M., Gill A. (2023). Evaluation of DNA extraction methods for the detection of Shiga toxin producing *Escherichia coli* in food by polymerase chain reaction. Int. J. Food Microbiol..

[62] Andrews S. (2010). Babraham Bioinformatics-FastQC a Quality Control Tool for High Throughput Sequence Data. https://www.bioinformatics.babraham.ac.uk/projects/fastqc.

[63] Bolger A.M., Lohse M., Usadel B. (2014). Trimmomatic: A flexible trimmer for Illumina sequence data. Bioinformatics.

[64] Meyer F., Paarmann D., D’Souza M., Olson R., Glass E.M., Kubal M., Paczian T., Rodriguez A., Stevens R., Wilke A. (2008). The metagenomics RAST server–a public resource for the automatic phylogenetic and functional analysis of metagenomes. BMC Bioinform..

[65] Lindgreen S., Adair K.L., Gardner P.P. (2016). An evaluation of the accuracy and speed of metagenome analysis tools. Sci. Rep..

[66] Keegan K.P., Glass E.M., Meyer F. (2016). MG-RAST, a metagenomics service for analysis of microbial community structure and function. Microbial Environmental Genomics (MEG).

[67] Dhariwal A., Chong J., Habib S., King I.L., Agellon L.B., Xia J. (2017). MicrobiomeAnalyst: A web-based tool for comprehensive statistical, visual and meta-analysis of microbiome data. Nucleic Acids Res..

[68] Segata N., Izard J., Waldron L., Gevers D., Miropolsky L., Garrett W.S., Huttenhower C. (2011). Metagenomic biomarker discovery and explanation. Genome Biol..

[69] Zeineldin M., Megahed A., Burton B., Blair B., Aldridge B., Lowe J.F. (2019). Effect of single dose of antimicrobial administration at birth on fecal microbiota development and prevalence of antimicrobial resistance genes in piglets. Front. Microbiol..

[70] Collado N., Buttiglieri G., Marti E., Ferrando-Climent L., Rodriguez-Mozaz S., Barceló D., Comas J., Rodriguez-Roda I. (2013). Effects on activated sludge bacterial community exposed to sulfamethoxazole. Chemosphere.

[71] Dutil L., Irwin R., Finley R., Ng L.K., Avery B., Boerlin P., Bourgault A.M., Cole L., Daignault D., Desruisseau A. (2010). Ceftiofur resistance in Salmonella enterica serovar Heidelberg from chicken meat and humans, Canada. Emerg. Infect. Dis..

[72] Rafii F., Williams A.J., Park M., Sims L.M., Heinze T.M., Cerniglia C.E., Sutherland J.B. (2009). Isolation of bacterial strains from bovine fecal microflora capable of degradation of ceftiofur. Vet. Microbiol..

