# Supplementary material for: Impact of Parenteral Ceftiofur on Developmental Dynamics of Early Life Fecal Microbiota and Antibiotic Resistome in Neonatal Lambs
Source: Antibiotics (Basel). 2025 Apr 25;14(5):434. doi: 10.3390/antibiotics14050434 (PMC12108499; doi:10.3390/antibiotics14050434)
Supplement: Supplementary file 1 [file antibiotics-14-00434-s001.zip › antibiotics-3586328-supplementary.pdf]

Table S1: Taxonomic classification of the fecal microbiome at the phylum level for both control and Ceftiofur (CCFA) treated lambs at each sampling time point (day 0, day 7, day 14, day 28, and day 56). This table summarizes bacterial phyla of relative abundance averaged more than 0.25% across all samples.

		Day 0			Day 7			Day 14			Day 28			Day 56		
		CCFA	control	p - value	CCFA	control	p - value	CCFA	control	p - value	CCFA	control	p - value	CCFA	control	p - value
Firmicutes	mean	0.62191	0.58897		0.68325	0.52353		0.31288	0.31021		0.51983	0.49301		0.62437	0.50584	
	SD ±	0.29281	0.17832	0.8336	0.13340	0.16511	0.0929	0.10524	0.06445	1	0.18234	0.15606	0.8336	0.07523	0.09333	0.0157
Bacteroidetes	mean	0.06237	0.04273		0.23418	0.28949		0.45783	0.46079		0.27097	0.27955		0.18660	0.24767	
	SD ±	0.17161	0.11387	0.5995	0.14393	0.22557	0.8336	0.13689	0.09364	1	0.13285	0.07805	0.8336	0.10310	0.10682	0.2936
Proteobacteria	mean	0.30996	0.35013		0.06989	0.17084		0.20881	0.20151		0.17811	0.18889		0.09819	0.16827	
	SD ±	0.28912	0.19364	0.5286	0.04410	0.11733	0.0742	0.09257	0.11695	0.9164	0.15728	0.14804	0.9164	0.04250	0.08605	0.0929
Actinobacteria	mean	0.00081	0.01098		0.00496	0.00636		0.01316	0.01540		0.01430	0.01504		0.04092	0.03814	
	SD ±	0.00047	0.00983	0.0008	0.00313	0.00429	0.5995	0.01424	0.01624	0.6674	0.00488	0.00762	0.6744	0.02323	0.03907	0.2936
Spirochaetes	mean	0.00027	0.00084		0.00100	0.00137		0.00085	0.00149		0.00196	0.00325		0.02327	0.00875	
	SD ±	0.00026	0.00180	0.8312	0.00126	0.00121	0.3446	0.00062	0.00077	0.0929	0.00121	0.00212	0.248	0.03127	0.00796	0.4008
Fusobacteria	mean	0.00076	0.00103		0.00101	0.00166		0.00114	0.00222		0.00274	0.00398		0.00471	0.00539	
	SD ±	0.00070	0.00127	0.6744	0.00093	0.00149	0.4622	0.00044	0.00106	0.0357	0.00166	0.00225	0.248	0.00191	0.00173	0.4008
Verrucomicrobia	mean	0.00001	0.00010		0.00019	0.00087		0.00145	0.00309		0.00294	0.00219		0.00244	0.00497	
	SD ±	0.00003	0.00014	0.046	0.00021	0.00179	0.3883	0.00252	0.00406	0.3446	0.00325	0.00283	0.7527	0.00261	0.00394	0.046
Cyanobacteria	mean	0.00109	0.00144		0.00116	0.00115		0.00062	0.00091		0.00154	0.00219		0.00303	0.00301	
	SD ±	0.00086	0.00099	0.5286	0.00095	0.00042	0.1722	0.00022	0.00031	0.0742	0.00085	0.00107	0.1722	0.00076	0.00108	0.6744
Chloroflexi	mean	0.00013	0.00028		0.00033	0.00061		0.00038	0.00048		0.00096	0.00133		0.00147	0.00183	
	SD ±	0.00016	0.00048	0.6698	0.00048	0.00059	0.2008	0.00012	0.00026	0.4008	0.00039	0.00078	0.3446	0.00087	0.00086	0.6744
Thermotogae	mean	0.00010	0.00019		0.00026	0.00051		0.00031	0.00057		0.00086	0.00141		0.00155	0.00190	
	SD ±	0.00012	0.00030	0.7422	0.00031	0.00051	0.3594	0.00020	0.00030	0.0587	0.00058	0.00100	0.248	0.00074	0.00086	0.4008
Synergistetes	mean	0.00008	0.00027		0.00022	0.00035		0.00029	0.00040		0.00068	0.00110		0.00157	0.00167	
	SD ±	0.00014	0.00060	0.5628	0.00030	0.00032	0.3938	0.00021	0.00028	0.6744	0.00044	0.00067	0.1275	0.00099	0.00088	0.7527
Chlorobi	mean	0.00039	0.00038		0.00066	0.00051		0.00044	0.00048		0.00046	0.00125		0.00162	0.00158	
	SD ±	0.00031	0.00030	1	0.00026	0.00022	0.248	0.00021	0.00017	0.6744	0.00030	0.00094	0.0357	0.00066	0.00055	0.7527
Tenericutes	mean	0.00006	0.00005		0.00020	0.00027		0.00025	0.00047		0.00072	0.00139		0.00129	0.00152	
	SD ±	0.00007	0.00013	0.216	0.00018	0.00027	0.7509	0.00017	0.00028	0.1152	0.00066	0.00090	0.1152	0.00069	0.00073	0.4622
Fibrobacteres	mean	0.00006	0.00013		0.00011	0.00020		0.00014	0.00027		0.00043	0.00076		0.00130	0.00139	
	SD ±	0.00012	0.00033	0.7017	0.00017	0.00016	0.2729	0.00012	0.00014	0.0585	0.00033	0.00060	0.2936	0.00075	0.00061	0.9164
others/ unassigned	mean	0.00199	0.00247		0.00260	0.00227		0.00145	0.00172		0.00352	0.00467		0.00767	0.00808	
	SD ±	0.00117	0.00084	0.3446	0.00076	0.00095	0.2076	0.00056	0.00064	75	0.00154	0.00335	0.8336	0.00316	0.00310	0.8336

Table S2: Taxonomic classification of the fecal microbiome at the phylum level for both control and Ceftiofur (CCFA) treated lambs at each sampling time point (day 0, day 7, day 14, day 28, and day 56). All other bacterial phyla of relative abundance averaged less than 0.25% across all samples are included in this table.

		Day 0			Day 7			Day 14			Day 28			Day 56		
		CCFA	control	p - value	CCFA	control	p - value	CCFA	control	p - value	CCFA	control	p - value	CCFA	control	p - value
Lentisphaerae	mean	3.76E 06	3.17E 05	0.927	2.53E 05	4.25E 05	0.469	4.09E 05	6.86E 05	0.6698	0.000179	0.00058	0.2698	0.001263	0.001379	0.9164
	SD ±	1.06E 05	8.96E 05		5.09E 05	6.86E 05		5.13E 05	8.30E 05		0.00018	0.00081		0.001389	0.001229	
unclassified (derived from Bacteria)	mean	0.001878	0.001812	1	0.002125859	0.001332	0.1152	0.000769	0.000407	0.5286	0.001306	0.000599	0.1722	0.001488	0.001242	0.6744
	SD ±	0.001299	0.001092		0.001028669	0.001179		0.000679	0.000159		0.00141	0.00027		0.000817	0.000365	
Acidobacteria	mean	7.53E 06	0.000116	0.4411	0.000109165	0.000204	0.0946	0.000154	0.000297	0.0742	0.000346	0.000653	0.1152	0.000756	0.000826	0.6744
	SD ±	2.13E 05	0.000216		0.000193617	0.000133		0.000117	0.000164		0.00019	0.000361		0.000384	0.000453	
Planctomycetes	mean	1.92E 05	0.000151	0.0821	6.69E 05	0.000105	0.4659	0.000125	0.000136	0.6742	0.000264	0.000685	0.248	0.000746	0.000915	0.4008
	SD ±	2.76E 05	0.000161		0.000103674	0.000103		0.000118	7.29E 05		0.000199	0.000776		0.000722	0.000564	
Deinococcus-Thermus	mean	0	9.69E 05	0.0645	7.85E 05	0.000151	0.3343	7.88E 05	0.000187	0.341	0.000291	0.000447	0.4622	0.000809	0.000774	0.8336
	SD ±	0	0.000213		0.000155114	0.000181		5.42E 05	0.000175		0.000168	0.000372		0.00034	0.000295	
Aquificae	mean	2.21E 05	2.51E 05	0.8903	2.66E 05	9.47E 05	0.2275	4.07E 05	0.000146	0.055	0.000205	0.000384	0.1415	0.00065	0.000683	0.6744
	SD ±	4.36E 05	4.76E 05		5.22E 05	0.000131		4.77E 05	0.000125		0.000176	0.000266		0.00024	0.000343	
Deferribacteres	mean	1.13E 05	7.07E 05	0.5377	1.80E 05	7.52E 05	0.0826	3.94E 05	0.000126	0.0156	0.000187	0.000314	0.1559	0.000537	0.000568	0.8336
	SD ±	3.19E 05	0.000169		3.33E 05	8.05E 05		3.67E 05	6.98E 05		0.000199	0.000229		0.000223	0.000178	
Elusimicrobia	mean	1.20E 05	1.91E 05	0.7826	5.06E 05	6.14E 05	0.5463	4.11E 05	8.32E 05	0.241	0.000179	0.000368	0.3431	0.000483	0.000567	0.7527
	SD ±	2.31E 05	3.61E 05		0.000101757	8.85E 05		5.45E 05	7.24E 05		0.000167	0.000334		0.000331	0.000387	
Dictyoglomi	mean	3.69E 05	8.44E 05	0.3983	4.46E 05	0.000109	0.2388	5.99E 05	0.000105	0.1412	0.000181	0.000286	0.3148	0.000299	0.00036	0.5992
	SD ±	6.98E 05	0.000113		6.87E 05	0.00011		5.73E 05	4.98E 05		0.000171	0.000216		0.000278	0.000199	
Chlamydiae	mean	0	1.72E 05	0.1441	3.13E 05	3.17E 05	0.7984	1.64E 05	5.89E 05	0.1248	8.63E 05	0.000125	0.3938	0.000255	0.000306	0.6742
	SD ±	0	3.27E 05		7.28E 05	6.96E 05		2.57E 05	6.01E 05		8.90E 05	0.000124		0.000225	0.000184	
Nitrospirae	mean	3.76E 06	6.93E 06	0.9273	0	2.60E 05	0.0273	3.18E 05	4.82E 05	0.661	8.71E 05	0.000129	0.2666	0.000135	0.00023	0.0734
	SD ±	1.06E 05	1.96E 05		0	3.11E 05		3.83E 05	5.36E 05		9.06E 05	6.19E 05		0.000142	7.23E 05	
Chrysiogenetes	mean	0	2.80E 05	0.1441	9.98E 06	2.12E 05	0.5232	3.94E 05	2.50E 05	0.9127	4.24E 05	5.55E 05	0.4916	0.000157	0.000128	0.4306
	SD ±	0	5.20E 05		2.42E 05	3.72E 05		7.11E 05	2.76E 05		3.77E 05	3.87E 05		0.000233	8.14E 05	
Gemmatimonadetes	mean	0	1.29E 05	0.3173	0.000002664	1.31E 05	0.4411	1.16E 05	1.77E 05	0.2472	0.000136	3.58E 05	1	7.08E 05	6.93E 05	0.8739
	SD ±	0	3.64E 05		7.53E 06	2.46E 05		2.68E 05	1.96E 05		0.000314	5.36E 05		7.63E 05	4.70E 05	
Candidatus Poribacteria	mean	0	0	1	8.65E 06	8.82E 06	0.9273	0	1.49E 05	0.0273	3.22E 05	8.27E 06	0.817	1.98E 05	3.26E 05	0.2729
	SD ±	0	0		2.45E 05	2.49E 05		0	2.01E 05		4.88E 05	1.07E 05		3.39E 05	2.70E 05	

Continued Table S2: Taxonomic classification of the fecal microbiome at the genus level for the control (control) and Ceftiofur (CCFA) treated lambs at each sampling time day (day 0, day 7, day 14, day 28, and day 56). Only those bacterial genera that averaged more than 0.25% of the relative abundance across all samples are displayed.

		day 0			day 7			day 14			day 28			day 56		
Analysis Columns	Statistics	CCFA	Control	P - value	CCFA	Control	P - value	CCFA	Control	P - value	CCFA	Control	P - value	CCFA	Control	P - value
Bacteroides	Mean	0.060659475	0.04056415	0.7527	0.22788525	0.2833213	0.8336	0.444301263	0.441031838	1	0.2500117	0.2162116	0.2936	0.130665288	0.180414638	0.248
	Std Dev±	0.167927742	0.110910112		0.140837968	0.221722315		0.131356872	0.084397947		0.125536602	0.089083597		0.062748693	0.095142782	
Clostridium	Mean	0.048931213	0.065818025	0.3446	0.064400863	0.077441463	0.2936	0.052642125	0.066427213	0.1415	0.0680031	0.089493788	0.248	0.09162275	0.103316363	0.6744
	Std Dev±	0.077504135	0.072368256		0.095792599	0.053239849		0.046600507	0.026190828		0.037896563	0.046035593		0.046763159	0.045070016	
Escherichia	Mean	0.219222338	0.127936238	0.6744	0.0314133	0.103352525	0.0929	0.161151025	0.136129838	0.5286	0.1190391	0.13118625	0.9164	0.016777063	0.046450025	0.1722
	Std Dev±	0.223571923	0.133883202		0.036339156	0.096974752		0.077202903	0.113010658		0.143938038	0.133320666		0.012346559	0.050377453	
Eubacterium	Mean	0.311692988	0.260800013	0.5286	0.308538363	0.192203288	0.0742	0.113304313	0.062509813	0.2076	0.161841825	0.090710625	0.0929	0.176579763	0.106660288	0.2936
	Std Dev±	0.193168848	0.132092297		0.133704143	0.136944049		0.081150219	0.023326021		0.127918872	0.034752752		0.109701622	0.056068109	
Streptococcus	Mean	0.247739338	0.219988025	0.6744	0.250550838	0.147289975	0.0587	0.089081413	0.047724488	0.2076	0.130237563	0.056659838	0.0742	0.134990138	0.079554713	0.2936
	Std Dev±	0.148651767	0.128566503		0.114708135	0.110997449		0.067012357	0.022975651		0.126904081	0.035796429		0.102861721	0.052708836	
Lactobacillus	Mean	0.0004316	0.0007595	0.3431	0.005888838	0.004379638	0.5286	0.007587263	0.0278078	0.2936	0.018032125	0.068234088	0.9164	0.03450885	0.018753925	0.1415
	Std Dev±	0.000555923	0.00083646		0.012631857	0.005736876		0.009830222	0.041278015		0.019522616	0.107970114		0.024356711	0.018113432	
Ruminococcus	Mean	0.002166938	0.0071977	0.5286	0.006722275	0.02237545	0.1722	0.005874563	0.01328125	0.046	0.020288475	0.028024963	0.2076	0.024575475	0.028298538	0.6744
	Std Dev±	0.003664912	0.016155044		0.008111084	0.030970999		0.004280268	0.006357984		0.01455628	0.01431037		0.015178015	0.014915455	
Prevotella	Mean	0.000717138	0.00051685	0.8335	0.001523938	0.001607988	0.9164	0.0029177	0.002920088	0.9164	0.0044795	0.0134116	0.1722	0.02094215	0.017948413	0.6744
	Std Dev±	0.001454457	0.000794643		0.000942214	0.001233818		0.00138522	0.000930181		0.005551144	0.016063129		0.019614145	0.010375439	
Shigella	Mean	0.026230863	0.015581288	0.5286	0.003227225	0.011804663	0.0587	0.018899563	0.015774875	0.5286	0.013866638	0.015774913	0.8336	0.002035013	0.00524725	0.3446
	Std Dev±	0.027133435	0.01809545		0.003787721	0.011315553		0.009496633	0.012868241		0.017110209	0.015862612		0.001537104	0.005824278	
Campylobacter	Mean	0.0000164	0.000170038	0.0491	0.00018205	0.01943855	0.5274	0.000169613	0.014527313	0.0742	0.001684625	0.00301695	0.4622	0.027705125	0.0531154	0.7527
	Std Dev±	3.05E 05	0.000156927		0.000161442	0.054478857		9.54E 05	0.04027263		0.003323505	0.006325595		0.035291818	0.072145325	
Parabacteroides	Mean	0.000568025	0.000390263	0.7017	0.002791463	0.002288813	0.6744	0.0066425	0.01002625	0.9164	0.006973375	0.017061988	0.046	0.007261113	0.00849945	0.4008
	Std Dev±	0.001558499	0.000956929		0.001925825	0.001554292		0.004084791	0.013597523		0.004479401	0.010882125		0.005758992	0.00418825	
Klebsiella	Mean	0.017419375	0.149753625	0.0454	0.000290275	0.0033946	0.0274	0.000920813	0.001651213	0.7527	0.00077645	0.000705763	0.6744	0.00026635	0.00054895	0.4622
	Std Dev±	0.04747931	0.203645654		0.000250579	0.005415278		0.000285233	0.002431856		0.000818052	0.00063665		0.00014005	0.000547696	
Bacillus	Mean	0.001261225	0.002221925	0.4622	0.001929938	0.00359535	0.3446	0.001871225	0.0037932	0.0357	0.007178938	0.011601313	0.1722	0.0097111	0.0109439	0.5995
	Std Dev±	0.001380854	0.002795253		0.001464739	0.003194314		0.000988059	0.00177386		0.005511448	0.007264761		0.004242075	0.004215353	
Roseburia	Mean	0.0002534	0.000719563	0.9151	0.00113015	0.00240815	0.0929	0.001439538	0.002897825	0.1722	0.008453625	0.009441025	0.4622	0.0075315	0.008611813	0.6744
	Std Dev±	0.000271356	0.001497284		0.001279698	0.001622162		0.001119222	0.00202893		0.011575554	0.008349779		0.005120342	0.005629095	
Bifidobacterium	Mean	0.000170788	0.00035595	0.7285	0.000982863	0.00150655	1	0.00191475	0.00281695	0.9164	0.001994363	0.003762525	0.248	0.009281713	0.017782625	0.8336
	Std Dev±	0.000250765	0.000672019		0.000560217	0.001801876		0.001784797	0.003165242		0.001360819	0.002933904		0.014005386	0.037021407	
Erysipelotrichaceae	Mean	0.000895563	0.000932588	0.7509	0.003239713	0.005904288	0.7527	0.002884363	0.00873005	0.0033	0.0102523	0.009368338	0.8336	0.005044713	0.0051403	0.5286
	Std Dev±	0.001574189	0.001894398		0.001822722	0.005884227		0.001544863	0.005892065		0.009874299	0.006237856		0.001492011	0.001804453	
Clostridiales	Mean	0.000248125	0.001266475	0.5107	0.003516775	0.007623825	0.046	0.007595688	0.008905913	0.5286	0.003485488	0.003357738	0.9164	0.00357675	0.003971938	0.4622
	Std Dev±	0.000436466	0.002922032		0.005110039	0.003736987		0.005788245	0.004122487		0.002803316	0.001801034		0.001281186	0.001282712	
Ruminococcaceae	Mean	0.000261688	0.002645588	0.5628	0.00126335	0.002368463	0.1719	0.001089675	0.002215025	0.0587	0.005153213	0.007949138	0.2936	0.007315188	0.007734575	0.7527
	Std Dev±	0.000374161	0.006833202		0.002534454	0.002151145		0.001054433	0.001448054		0.004850632	0.005260237		0.005157498	0.004340217	
Butyrivibrio	Mean	0.000172375	0.000658813	0.2472	0.000859538	0.0015016	0.2933	0.000840913	0.00182325	0.1152	0.004322213	0.0081549	0.1722	0.00916065	0.01048965	0.7527
	Std Dev±	0.0003722	0.001257653		0.001041855	0.001315503		0.000524754	0.001102968		0.003711983	0.006053948		0.006165306	0.007093546	
Neisseria	Mean	0.026544775	0.0228153	0.7527	0.023974613	0.014621213	0.0929	0.009519438	0.004343088	0.2076	0.012516838	0.005540713	0.0742	0.012669075	0.007409638	0.4008
	Std Dev±	0.016316727	0.013643695		0.011470463	0.011118378		0.008229908	0.00200878		0.012859783	0.003381047		0.009976115	0.005933572	
Faecalibacterium	Mean	0.000116838	0.001121563	0.3883	0.001176125	0.002569438	0.0919	0.0017528	0.006229525	0.1152	0.005728075	0.005811825	0.5286	0.005162563	0.005871475	0.5286
	Std Dev±	0.000135245	0.00236618		0.001806964	0.002588884		0.002011799	0.006878136		0.006264701	0.003005834		0.003157638	0.002459084	
Blautia	Mean	0.000216213	0.00105025	0.3374	0.00125925	0.0031668	0.0742	0.0013085	0.0031107	0.0587	0.005869563	0.007425463	0.4622	0.00473155	0.00629045	0.4622
	Std Dev±	0.000285601	0.002222223		0.001864814	0.003086871		0.001042268	0.002056145		0.005782191	0.005272206		0.003210152	0.004381867	
Alistipes	Mean	2.14E 05	0.00001525	0.644	0.000146825	0.000079575	0.7059	0.000142975	0.001447488	0.0404	0.003117075	0.007905975	0.2076	0.007318088	0.009598463	0.248
	Std Dev±	6.06E 05	3.05E 05		0.000186912	7.53E 05		0.000149694	0.002432396		0.003559433	0.009300294		0.007783825	0.00573811	
Ethanoligenens	Mean	0.000195325	0.001822213	0.5224	0.001761413	0.004179763	0.4622	0.001317863	0.002612813	0.1152	0.002996475	0.004868938	0.2076	0.0054559	0.0054028	0.7527
	Std Dev±	0.00024154	0.004278082		0.003219706	0.00429973		0.001271202	0.00176853		0.002401147	0.003458294		0.003762141	0.00289838	
Anaerotruncus	Mean	0.000101975	0.00132015	0.817	0.001983563	0.00525925	0.5606	0.001655375	0.003778463	0.248	0.001682075	0.002709788	0.2836	0.0030597	0.0031049	1
	Std Dev±	0.000140201	0.002522053		0.004130695	0.006846114		0.002061392	0.00556782		0.001260317	0.001981447		0.001805587	0.001676972	
Anaerococcus	Mean	6.72E 05	0.000235988	0.5628	0.000312225	0.0004294	0.6721	0.000550788	0.001772763	0.2076	0.005411313	0.004780813	0.5286	0.015645175	0.007362575	0.8336
	Std Dev±	0.000116061	0.000491394		0.000354774	0.000419606		0.000676822	0.003125671		0.003891288	0.004817749		0.018702985	0.004307739	
Salmonella	Mean	0.008197625	0.010111275	0.4008	0.001058375	0.004271125	0.0357	0.004424938	0.004412238	0.9164	0.003644425	0.0036365	1	0.000696925	0.001849713	0.0742
	Std Dev±	0.009085921	0.006792484		0.001224825	0.004618477		0.001672475	0.003271183		0.003979627	0.003433157		0.000391152	0.001583523	
Enterococcus	Mean	0.0003323	0.002054113	0.1275	0.00789605	0.005681713	0.1722	0.001619338	0.0022737	0.1722	0.0031888	0.002470875	0.9164	0.002967788	0.003379713	0.7527
	Std Dev±	0.000375963	0.003095105		0.005575212	0.008127873		0.000938512	0.000826032		0.002942003	0.001001982		0.001428397	0.001991986	
Treponema	Mean	8.03E 05	0.000154763	0.203	0.000270363	0.000418738	0.5274	0.000191638	0.00047715	0.0156	0.000698213	0.001268613	0.1722	0.017528175	0.005423688	0.4622
	Std Dev±	8.71E 05	0.000405466		0.000298266	0.000396974		0.000170566	0.000220205		0.000485772	0.00092274		0.026276145	0.005980856	
Desulfovibrio	Mean	4.41E 05	0.00016475	0.4875	0.000325375	0.0005173	0.2237	0.002408438	0.006406275	0.1415	0.004220125	0.004632938	0.5995	0.003192913	0.005283338	0.1722
	Std Dev±	6.83E 05	0.000327163		0.000411856	0.000446557		0.003471231	0.007965245		0.003114533	0.002108354		0.001569642	0.00341938	
Holdemania	Mean	0.000284963	0.000821763	0.5283	0.001496288	0.002207425	0.4008	0.001253963	0.003687888	0.0209	0.003444288	0.004518213	0.4622	0.003632325	0.003818163	0.6744
	Std Dev±	0.000287622	0.001487327		0.001106868	0.001641454		0.000825841	0.002396445		0.002224063	0.0024258		0.00158944	0.001718151	
Others	Mean	0.024738	0.06003595	0.0117	0.04200245	0.06279165	0.2076	0.052725763	0.088453675	0.0587	0.111408125	0.160302	0.2936	0.198389238	0.221722475	0.4008
	Std Dev±	0.01449064	0.036692468		0.027537499	0.039325571		0.023822295	0.041734135		0.047258991	0.08654535		0.066019488	0.068206019	

Table S2: Summary table of the significant microbiome changes in CCFA-treated lambs over time with supporting evidence from the literature.

	Time point	Significant changes in CCFA-treated	Literature key points	References
Phylum level	Day 14	↓ *Fusobacteria*	Similar decrease in pigs	[1]
	Day 28	↓ *Chlorobi*	Similar reduction in complex environments treated with antimicrobials.	[2]
	Day 56	↓ *Firmicutes*	Contradictory results in pigs	[3]
Genus level	Day 7	↓ *Salmonella* unclassified derived *Clostridiales*	Similar results in pigs and chickens	[4, 5]
	Day 14	↓ *Bacillus*	Can degrade the ceftiofur.	[6]
		Ruminococus *Holdemania* Alistipes	Are important for cellulose digestion and rumen development.	
		Treponema *Erysipelotrichaceae*	Contradictory reduction in pigs with single dose of ceftiofur	[1]

1. Zeineldin, M., et al., *Effect of single dose of antimicrobial administration at birth on fecal microbiota development and prevalence of antimicrobial resistance genes in piglets.* Frontiers in microbiology, 2019. **10**: p. 1414.
2. Collado, N., et al., *Effects on activated sludge bacterial community exposed to sulfamethoxazole.* Chemosphere, 2013. **93**(1): p. 99-106.
3. Foster, D.M., et al., *Ceftiofur formulation differentially affects the intestinal drug concentration, resistance of fecal Escherichia coli, and the microbiome of steers.* PLoS One, 2019. **14**(10): p. e0223378.

4. Dutil, L., et al., *Ceftiofur resistance in Salmonella enterica serovar Heidelberg from chicken meat and humans, Canada.* Emerging infectious diseases, 2010. **16**(1): p. 48.
5. Rutjens, S., et al., *Intestinal Exposure to Ceftiofur and Cefquinome after Intramuscular Treatment and the Impact of Ceftiofur on the Pig Fecal Microbiome and Resistome.* Antibiotics, 2022. **11**(3): p. 342.
6. Rafii, F., et al., *Isolation of bacterial strains from bovine fecal microflora capable of degradation of ceftiofur.* Veterinary microbiology, 2009. **139**(1-2): p. 89-96.
